# The Fourth Venereal Disease

**Published:** 1913-10

**Authors:** 


### ABSTRACTS.

*Readers are invited to assist in this department. With rare exceptions, we avoid abstracting from journals of organizations to which most of our readers belong. Whenever available, we state the full name and address of authors and the name and date of the journal of original publication.*

THE FOURTH VENEREAL DISEASE. Some four years ago Corbus and Harris reported observations which tended to confirm the claims of Scherber and Mueller that a fourth distinct venereal disease existed. According to Corbus, the condition is a specific balanitis, and is identical with the *balanoposthite érosive circinée* of Bataille and Berdal, the latter representing a more aggravated stage of the disorder, but having the same bacteriologic genesis.

The infection is due to a symbiosis of a spirochete and a vibrio. Corbus sees a distinct etiologic influence in the gratification of the sexual impulse through unnatural means. Practically all of his patients confessed to unnatural practices. It is thought that certain organisms transferred from the buccal cavity to a more favorable habitat beneath a long, tight foreskin, may take on a luxuriant growth with a marked increase in pathogenic power. Thus the spirochete found in Vincent's angina in repeated examinations was determined to be identical with the spirochete of erosive and gangrenous balanitis, a marked feature being its motility.

A clinical characteristic of the development of the erosion is the production of a thin, offensive purulent secretion exuding from beneath the prepuce. Of course, to differentiate the condition from gonorrhea it is but necessary to determine the origin of the pus. As a rule the infection rapidly subsides, if in the erosive stage, by cleansing the surfaces and thereafter maintaining a state of cleanliness.

